# General practitioners as educators in adolescent health: a training evaluation

**DOI:** 10.1186/s12875-016-0432-0

**Published:** 2016-03-22

**Authors:** Thea Van de Mortel, Jennifer Bird, Peter Chown, Robert Trigger, Christine Ahern

**Affiliations:** School of Nursing & Midwifery, Griffith University, Parklands Drive, Southport, QLD 4215 Australia; Birdtalk, 39 Leslie Street, Bangalow, 2479 NSW Australia; Bangalow Professional Centre, Suite 4, Lot 1 Ballina Road, Bangalow, NSW 2479 Australia; North Coast GP Training, PO Box 4978, Ballina, NSW 2478 Australia

**Keywords:** Adolescents, Community health education, Medical education, Training, General practitioners, Evaluation

## Abstract

**Background:**

General practitioners play an important role in the primary care of adolescents in both community and clinical settings. Yet studies show that GPs can lack confidence, skills and knowledge in adolescent health. This study evaluates the effectiveness of an innovative training intervention on medical participants’ knowledge and confidence as adolescent health educators in a school setting.

**Methods:**

15 general practitioners, 12 general practice registrars and 18 medical students participated in an adolescent health education workshop followed by field experience in health education sessions in secondary schools. The mixed method design included a pre and post intervention survey and focus group interviews.

**Results:**

Mean scores on the Confidence to Teach scale increased significantly (3.34 ± 0.51 to 4.09 ± 0.33) (*p* < .001) as did confidence to communicate with adolescents (3.64 ± 0.48 to 4.19 ± 0.33) (*p* < .001). Mean knowledge scores increased significantly (7.00 ± 1.22 to 8.98 ± 1.11) (*p* < .001). Participants highlighted the value of learning about adolescent health issues and generic teaching skills especially lesson planning and design, practicing experiential teaching strategies and finding the ‘sweet spot’ when communicating with adolescents. Some participants reported that these skills would transfer to the practice setting.

**Conclusion:**

An applied training intervention that uses evidence-based, experiential teaching strategies and focuses on developing knowledge and practical teaching skills appropriate for the health education of adolescents can enhance knowledge and confidence to engage in community-based adolescent health education.

## Background

The major health problems of adolescents are predominantly psychosocial and thus potentially preventable [1, 2]. Adolescence is a key period for the adoption of behaviours that are critical in shaping future health status, and GPs have a crucial role in the early detection of health risk behaviours and the provision of timely health education [3, 4]. However, there is a range of barriers to the provision of effective primary healthcare to young people. Lack of knowledge by young people of the GP’s role, difficulties accessing GPs and concerns about privacy and confidentiality [2, 5, 6] impact on the rates at which adolescents access GP services. In turn, GPs have identified inadequate training in adolescent health, leading to a lack of confidence, knowledge and skills in adolescent health issues, and difficulties in effectively engaging and communicating with young people [7–9].

Outreach programs have been identified as one model of ‘youth friendly’ service delivery where ‘health providers meet [adolescents] in settings in which they feel comfortable.’ [5]. In Australia there are a number ‘GPs in Schools’ outreach programs that aim to improve young people’s access to GPs and the provision of health education [10]. However, studies consistently report GPs have low efficacy in dealing with adolescents [11]. Additionally, whilst GPs are expected to engage in health promotion and health education activities [12, 13], they also lack confidence and skills in delivering community based health education [3, 14–18].

There is evidence that in the clinical setting providing training to GPs in adolescent health is effective in improving knowledge of adolescent health issues [7] and perceived confidence and competence in providing clinical services to adolescents [7]. Additionally, participating in health promotion workshops, completing community placements during undergraduate training, and gaining experience at addressing a community group can improve GP confidence and skills in delivering community based education [3, 14–18].

Engaging in outreach programs in the community, and in schools in particular, requires GPs to not only have sound knowledge of adolescent health issues but to be confident and competent in the skills of community-based adolescent health education. Some ‘GPs in Schools’ training interventions contain a component of health education skills [10, 19]. However, there are no reported training programs that focus on providing GPs with thorough theoretical and practical training in community based health education skills generally, and in particular the sub speciality of adolescent health education.

The training intervention evaluated in this study contained both a one-day workshop and an applied field experience in a school facilitating a health education session with Year 11 students. The intervention aimed to:

- Increase knowledge in adolescent health in order to better understand young people’s health issues and concerns
- Improve skills and confidence in communicating with young people
- Improve knowledge and skills in small group facilitation and experiential teaching strategies
- Increase knowledge and skills in planning and delivering a health education session.

The training intervention took a vertically integrated (VI) approach to participants and included a mixed group of general practitioners (GP), GP registrars (GPR) and medical students (MS). Previous research has demonstrated that the MS and GPR, who are closer in age to the school students, may be seen as more approachable by adolescents, which can help to create a bridge between older GPs and adolescents [10], while GPs may bring a greater level of knowledge of adolescent health issues. That study found that participants enjoyed and learned from the ‘mix’ of knowledge and skills in both adolescent health and teaching strategies for adolescents amongst and between the three levels GP, GPR and MS.

The aim of this study was to evaluate self-reported impacts of the training intervention on the participants’ knowledge, skills and confidence in delivering adolescent community based health education.

## Methods

### Participants and setting

A purposive sample of GPs, GPRs and MSs was recruited via email and electronic newsletter. The sampling aimed to recruit sufficient GPs, GPRs and MSs to form integrated teams that could deliver health education sessions to six or seven small groups at each secondary school. Information was provided on the study aims, requirements, and participants’ rights. The intervention was conducted in the Northern Rivers and Mid North Coast regions of NSW, Australia between August and October 2013. Participants were remunerated for their time and travel costs as part of the intervention occurred during surgery hours and considerable travel was involved to the regional locations.

### Training intervention design

The design of the workshop was informed by contemporary research findings about effective adolescent health education [20]. This research cautions against a singular focus on facts and knowledge in favour of functional health information; exploring and developing individual values and beliefs and group norms that support health behaviours; and developing skills that support health [20]. The workshop introduced participants to a range of active, participatory learning strategies commonly employed in adolescent health education, and the pedagogy that underpins them. Training techniques employed in the workshop included short didactic presentations, practicing skills, direct feedback, coaching, peer collaboration and small group discussions.

Brief didactic presentations were delivered to increase participant’s knowledge of adolescent health and factors that impact on it, including:

- Adolescent psychosocial development
- Major adolescent morbidity and health concerns
- The GP’s role in providing treatment and health education to young people
- Medico-legal issues in working with young people – especially confidentiality and privacy.

In order to support the development of skills in adolescent health education, the following presentations were delivered:

- Contemporary experiential, learner-centred teaching strategies employed in adolescent health education
- Communication and small group facilitation skills
- Planning a health education session.

An innovative feature was the use of young actors. Six young people from a local youth theatre group participated in the workshop in three different training techniques.

1. Role plays. The young actors underwent intensive training to prepare them to participate as “school students” in role-plays, which simulated the school environments in which the GPs would conduct their health education sessions. The young actors were trained not only to act as school students, but also in techniques for giving constructive feedback and coaching to the GPs in the role-plays. Numerous studies have identified the effectiveness of role-play techniques and simulated practice in enhancing the communication and consulting skills of doctors and other health professionals in the clinical setting [21–23].
2. Lesson planning. Participants collaborated in teaching teams to prepare lesson plans for a health education session in a local high school, providing participants with clear learning outcomes from the workshop that were then applied in an authentic context. One young actor was allocated to each teaching team and contributed to the design of the lesson plans and teaching strategies for the school health education sessions.
3. Panel discussion. The young actors discussed adolescent health issues and concerns with the group and fielded questions from the group about their perspectives on adolescent health and barriers and enablers to access to GP services.

### Study design

A mixed methods approach was used, which included a pre and post intervention survey, and qualitative data obtained via focus groups. A questionnaire was designed that contained some items validated in a previous study [10]. The previously used scales were adjusted and extended based on the pilot results. Cronbach’s alpha results are reported in the results section. The questionnaire was administered before and after a seven-hour workshop delivered at two locations. Participants were allocated to teaching teams. Each team consisted of either one GP, one registrar and one MS, or one GP and one or two MSs. Teams subsequently co-facilitated 90-min health education sessions at participating schools (teams visited 1–2 high schools in their area).

The following outcomes were assessed via the pre and post-intervention questionnaire:

- Self-reported perceptions of teaching skills and confidence to teach on a 9-item 5-point Likert scale.
- Self-reported confidence to communicate with adolescents on a 7-item 5-point Likert scale.
- Knowledge of adolescent health, medico-legal and general practice access issues via 10 multiple-choice questions.

Focus group semi-structured interviews were conducted at both locations within four weeks of the school visits to obtain qualitative feedback on the intervention. Participants were firstly asked to describe their experience of being a participant in the training workshop, what they thought was just right, what they would prefer less or more of, and whether they felt their knowledge or skills changed as a result of being a participant (and if so, how?). Subsequently, participants were asked what they felt worked well in relation to the school education sessions, what if anything they would change and why, if they felt their knowledge, skills or attitudes had changed as a result of the experience (and if so, how?). The focus groups were held in commercial buildings hired for the purpose at each study location. An interview schedule was used. Group sizes ranged from three to eight and the sessions lasted approximately one hour each. Separate focus groups were run for each level of medical participant to encourage free discussion of the experience, thus there were a total of six focus groups. With participant permission, focus group interviews were audio-recorded and transcribed verbatim. Transcripts were de-identified prior to data analysis. The post intervention questionnaires were completed at the beginning of each focus group.

### Ethics and permissions

Ethics approval was obtained from the Southern Cross University Human Research Ethics Committee (**ECN**-**13**-**084**). Written consent was obtained from each volunteer. Permission was obtained from each of four secondary schools to deliver health education sessions to their Year 11 students (aged ~16–17).

### Data analysis

Descriptive statistics were calculated on survey items. Cronbach’s alpha was used to determine scale reliability. A paired samples *t*-test examined differences in pre and post intervention survey scores. Two researchers independently conducted a thematic analysis of de-identified focus group transcripts using the steps described by Braun and Clark [24]. Codes and themes were discussed and agreement reached on the major themes. Thematic saturation was reached for some themes across all focus group data, with other themes remaining particular to either location or level of medical participant. Only major themes are reported in this study. Small sample sizes make invalid a comparative analysis of data either between locations or between levels of medical participants.

## Results

Forty-five volunteers participated: 15 GPs, 12 GPRs and 18 MSs. Twenty-seven were located in the Northern Rivers region of New South Wales (NSW) and 18 in the Mid North Coast region. Eighty percent were female (75 of GPs, 100 of GPRs and 72 % of MSs). Scale reliability on both the Confidence to Teach and Confidence with Adolescents scales was in the range considered good (Cronbach’s alpha 0.80). There was a 93 % response rate to the post intervention questionnaire. Appendix: Table 1 contains a summary, with examples, of the key themes and sub themes evident from the thematic analysis of the qualitative data.

### Knowledge of adolescent health issues

Participants’ mean scores on the knowledge quiz on adolescent health and general practice access issues increased significantly from 7.00 (±1.22) to 8.98/10 (±1.11) (t = −8.705; df = 41; *p* < .001). In the qualitative comments respondents indicated:

- Increased knowledge about Medicare (Australia’s national healthcare system) access, confidentiality and disclosure (medical students specifically).
- An increased awareness of adolescent concerns, for example mental health, drugs, self-harm, and issues of privacy and confidentiality for adolescents accessing primary care (See Appendix: Table 1. Themes from the focus group qualitative data).

For example:

*‘I think I have a better idea about the lack of knowledge that young people have about accessing the health care system. They don’t get taught about Medicare or about GPs in school and they just have to work it out for themselves.’* MS

### Confidence to engage with adolescents

There was a significant increase in confidence to consult with and communicate with adolescents from 3.64 (±0.48) to 4.19 (±0.33) (t = −7.196; df = 41; *p* < .001). Some participants indicated improved skills to communicate with adolescents and a better feeling for the ‘sweet spot’ for the language they would use (for example, avoiding jargon). All groups indicated that what they had learnt would change, or already had changed, the way they dealt with adolescents in their current practice. For example participants indicated that they would:

1. Listen more and talk less
2. Not make assumptions about adolescents’ knowledge of confidentiality, Medicare access and bulk billing
3. Find an appropriate level of language
4. Be better able to advise on issues such as getting a Medicare card.
5. Make a specific effort to discuss issues of confidentiality and disclosure up front.

For example,

‘*just reflecting on the last couple of days, the adolescent patients that I’ve seen, I’ve been [asking] ‘Oh, so do you have your own Medicare card, or are you just here with your family’s? Oh well, you can get that if you want’…whereas beforehand I think I made the assumption that if they’re here at the appointment that they’ve got their stuff sorted and they know how to do it, so I’ve sort of changed in that regard’* GPR

### Teaching skills and confidence to facilitate small groups

Participants’ mean scores on the Confidence to Teach scale increased significantly from 3.61 (±0.46) to 4.02 (±0.31) (t = −6.650; df = 41; *p* < .001). Participants reported that positive impacts of the workshop and/or school visits on their teaching skills included increased:

- confidence to teach and facilitate groups
- skill to design and plan a lesson using a lesson plan template, with consideration of timing, flexibility to meet different needs and circumstances, and co-facilitation skills
- knowledge and skill using a range of experiential teaching strategies appropriate to adolescent health education.

For example:

*‘I did a talk for students a couple of months before this workshop but I didn’t have the skills to deliver it to keep them interested and interacting, whereas now I’d use my same content but deliver it in a much, much different way. I would make it a lot more interactive and I would know what sort of techniques to use’* GP

Overall respondents reported greater school student engagement with experiential teaching strategies and reported that they were pleased that they had learned more about this pedagogical approach. Some reported that they would change their pedagogical approach to adolescent health education in the future as a result of this experience.

### The young actors

Participants valued the contribution of the young actors in the panel discussion of adolescent health issues, the role-plays of a simulated classroom, and their contribution to the development of lesson plans and teaching strategies for the school sessions.

*‘I think we needed the adolescents in front of us and we needed the group to practice.’* GP

*‘I particularly enjoyed listening to the adolescents and the feedback they provided… I guess in my work I don’t come across that particular age group and I found it really quite valuable to listen to what they had to say.’* GP

## Discussion

The findings from this small study suggest that an applied training intervention with medical professionals that focuses on developing knowledge and practical teaching skills appropriate for the health education of adolescents can enhance knowledge, confidence, and willingness to engage in community-based adolescent health education.

The study confirms previous findings [7, 21, 25] that well designed training interventions that use evidence-based teaching strategies can have positive impacts on doctors’ knowledge and self-efficacy in working with adolescents. Unlike previous studies, this study focused primarily on the development of community health education skills rather than clinical skills.

The study also extends existing knowledge about the use of young actors and role-plays in medical education for clinical settings into the new setting of the school classroom. In Australia, the use of young actors to train GPs towards improving their clinical practice skills with adolescents in particular has been reported by Sanci et al. [25] and Sanci et al. [3] who found an improvement in interviewing and communication skills, as well as self-perceived competency in working with adolescents. This study is different in that it builds on this evidence base and employs young actors in role-plays that simulate groups of adolescents in classroom settings rather than individuals in clinical settings.

The study raises interesting questions regarding the role of general practitioners in community-based health education activities in general and with adolescents as a population group in particular, and confirms findings from previous studies [16] that interventions such as this can improve the likelihood that general practitioners will engage in health promotion and health education activities in the community. The vertically integrated nature of the cohort in this intervention may offer a model for building confidence and skills across the medical education continuum.

However community based health education of this kind remains a minor component in the overall role of a general practitioner. Barriers that obstruct GPs playing a larger role in health promotion are considerable [14–18] and whilst an intervention such as this may reduce barriers to engagement at the individual practitioner level, responses to larger structural and practice barriers remain unexplored.

The study also highlights some differences between the skills required of doctors to fulfil their professional responsibilities as clinical teachers and the skills required to be community health educators of adolescents. Some participants in this study drew distinctions between aspects of the two, particularly in terms of the types of teaching strategies used to engage youth, although other generic teaching skills such as lesson planning were considered transferrable across contexts.

We did not aim to evaluate outcome and access impact for the participating adolescents, nor the degree to which the knowledge and skills gained transferred to clinical practice. Questions regarding sustainability and scalability were also not addressed in the evaluation. Whilst pre-post designs such as that employed in this study are not as strong as having a comparison group, the mixed method design offers multiple data sets, and the qualitative comments help triangulate the quantitative findings.

## Conclusion

This intervention supported GPs to extend their health education role out into the community, in this case into schools. Results from the study of the intervention both support and extend current research about effective training for the improvement of GPs knowledge about adolescent health and skills for clinical practice. It opens up opportunities for further research about the efficacy, efficiency and sustainability of GPs involvement in community health education in general, and in adolescent community health education in particular.

## Appendix

**Table 1:** Themes from the qualitative data

	Theme	Participant data
Knowledge of adolescent health issues	Access to general practice	*I have a better idea about the lack of knowledge that young people have about accessing the health care system.* GP
	Adolescent health issues	*to have a little bit of a feel for the statistics and what’s actually happening out there was valuable. I learned about self-harm and I learned about illicit drugs and that was information I didn’t have before. GP* *‘getting a feel for drugs (especially illicit drugs), mental health issues and self harm. Haven’t come across self- harm – bigger than I thought. GP*
Confidence to engage with adolescents	Listen	*When I consult with teenagers I will listen a lot more and try and get a feel for what they really want to talk about, rather than my thing. (GPR)*
	Don’t make assumptions about prior knowledge	*The one way it’s going to impact on me is to… take a step back with them and make sure that they understand what they’re talking about. (GPR)* *…the issues or concerns that young people have when they come to see a GP, things like really worrying about confidentiality, and now knowing that’ that’s something I have to reassure them about, first off. MS* *beforehand I just …made the assumption that if they’re here at the appointment that they’ve got their stuff sorted and they know how to do it, so I’ve sort of changed in that regard.’ GPR*
	Get the language right	*It helped get a good idea of where the ‘sweet spot’ is in terms of not dumbing it down too much, but not having too much jargon for that age group. MS*
	How to structure a consult	*I learnt things that I’ll be able to use in practice about engaging people MS* *the exposure to teaching young people has [given me] an idea of how I structure a consult. MS*
	Include adolescent health issues	*I am now making a more conscious effort to make sure that mental health issues are covered. GPR)*
Teaching skills & confidence to facilitate small groups	Confidence and enthusiasm	*I’m now more confident about teaching certainly. MS* *I came away wanting to learn more about all the different types of teaching methods. GPR* *I am more keen to teach. (GPR)* *I am more keen to teach…‘because it’s taken away a bit of the fear.’ GPR*
	Planning a lesson/using a lesson plan template	*I’m more confident in my ability to design a relatively structured lesson plan. MS* *I feel more well equipped to plan … and carry out a teaching session. GPR* *Planning definitely; different techniques; the template was helpful; better prepared, better equipped for it. (GPR)* *It’s great to have a tool to actually work it out… how you’re actually going to run this. GP*
	Teaching strategies for health education with adolescents	*It’s given me some tools to use rather than just feeling like right, well I need to get up there and tell them all I know.’ GPR* *I did come out of there with new strategies for myself for teaching that maybe the medical student might have used, or what other groups [used], that was really helpful to come back and talk about what worked and what didn’t work. GP* *I’ve tended to kind of just talk and talk and talk to people…[and now realise I need to] let there be more communication going on rather than that kind of didactic type of thing. GP* *I just learnt that engaging the kids it’s easier if they do self directed things, asking them questions and activities and stuff like that. MS*

## Abbreviations

GP: general practitioner
GPR: general practice registrar
MS: medical student
